# Engaging and Supporting Young Children and their Families in Early Childhood Mental Health Services: The Role of the Family Partner

**DOI:** 10.1007/s10597-021-00796-8

**Published:** 2021-02-28

**Authors:** Sameera S. Nayak, Carol Tobias, Jessica Wolfe, Kate Roper, Larisa Méndez-Peñate, Christy Moulin, Malika Arty, Arielle A. J. Scoglio, Amy Kelleher, Jacqueline Rue, Molly Brigham, Tarsha Bradshaw, Natasha Byars, Angelina Camacho, Sade Douglas, Beth E. Molnar

**Affiliations:** 1 Institute for Health Equity and Social Justice Research, Northeastern University, 360 Huntington Ave, Mail Stop 314 INV, Boston, MA 02115 USA; 2 Massachusetts Department of Public Health, Bureau of Family Health and Nutrition, Boston, MA USA; 3 Early Childhood and Family Mental Health Program, Boston Public Health Commission, Boston, MA USA; 4 Codman Square Health Center, Dorchester, MA USA; 5 Southwest Human Development, Phoenix, AZ USA

**Keywords:** Mental health, Early childhood, Lived experience, Family partner, Quality of care

## Abstract

This study explores the role of family partners, peer professionals with lived experiences of raising a child with behavioral health needs, and their value in primary and community-care based mental health services for young children aged 0–8 years. Interviews and focus groups were conducted with staff, leadership, and caregiver participants (n = 38) from two early childhood mental health programs and analyzed using thematic analysis. Five interdependent themes emerged: (1) the centrality of lived experience to the family partner role; (2) the importance of the family partner in family engagement and relationship building; (3) the value added by the family partner in navigating systems; (4) the ability of the family partner to build skills and empower caregivers; (5) the role of the family partner in alleviating caregiver stress and other mental health concerns. Adapting and expanding the role of family partners will improve effective mental health care for children and their caregivers.

I was stressed, like really stressed out until she [family partner] …came and everything just calmed down…a lot

- Caregiver

## Introduction

An estimated 9–14% of children between the ages of 0–5 years in the United States experience emotional and/or behavioral difficulties, which can have long-term adverse effects (Brauner & Stephens, 2006). Timely early childhood mental health (ECMH) intervention for these young children can be critically important for healthy development and to ensure future success in education, employment, and relationships (Boat et al., 2016). ECMH can be influenced by factors at all levels of the socio-ecological model (Bronfenbrenner, 1979). Individual and community-level factors affecting families such as homelessness, poverty, maltreatment, caregiver separation or loss, untreated mental health and substance use disorders, and other social and environmental determinants have been shown to negatively impact ECMH (Bayer et al., 2011; Garner & Shonkoff, 2012). At the same time, nurturing relationships with caregivers, and the early identification of behavioral difficulties with family-centered care can help mitigate these risks and promote positive mental health outcomes (Burak & Rolfes-Haase, 2018). Family-centered care is an approach that emphasizes collaborative decision-making and a partnership between patients and providers (Kuo et al., 2012). Peer support workers can be one avenue to better integrate family-centered care practices into ECMH.

A robust body of literature describes peer support workers and community health workers as core components of the mental health service delivery system in the U.S. (Barnett et al., 2018; Swider, 2002). More recently, the terms “experienced-based experts” and “peer support service providers” have been used to describe people who provide care coordination on health issues that they themselves have had lived experience with, such as mental health (Chinman et al., 2014; Davis et al., 2010; Repper & Carter, 2011). In Massachusetts, the role of a family partner has become an integral part of family-centered care. Family partners are a type of experience-based expert who combine peer support services with a family-centered approach, and who provide services drawing on their lived experience navigating systems and accessing services to meet their own child’s mental health needs. Family partners in Massachusetts became ubiquitous as part of the Children’s Behavioral Health Initiative (CBHI) which sought to provide a system of care for youth who receive Medicaid under the age of 21 and who suffer from behavioral, mental, and emotional difficulties. CBHI was formed in response to two important court cases, both highlighting that children on Medicaid were not given adequate evaluation and their needs were not met in their communities regarding their mental and social-emotional health (Rosie D. v. Romney, United States District Court, D. Massachusetts, 2006).

Family Partners help families access the right community services, provide peer support, advocate for the family, improve quality of services and transfer advocacy skills to caregivers (Gilkey et al., 2011; Pediatrics n.d). Studies have found that caregivers receiving peer support services reported improved self-care, empowerment and communication skills (Brister et al., 2012), more knowledge about symptoms and reduced stress (Jamison et al., 2017), less parental anxiety (Ireys et al., 2001), higher satisfaction with care, higher participation in services, and better social connectedness than those receiving care as usual (Radigan et al., 2014). Family partners have a unique role because of their lived experience, which allows them to form meaningful connections with families and serve as a trusted guide through a myriad of complex systems.

The LAUNCH/MYCHILD model of ECMH integration discussed in this article (described more fully below) pairs a dedicated team of a family partner and a mental health clinician in collaboration with primary care practices and community service agencies largely serving Medicaid patients. This model was implemented through two projects in Massachusetts—Project Linking Actions with Unmet Needs in Children's Health (LAUNCH) and the Massachusetts Multi-City Young Children's System of Care Project (SOC). An evaluation study of the first iteration of the Massachusetts LAUNCH project found that the family partner-clinician team model is efficacious in improving child social, emotional and behavioral problems and in reducing caregiver stress and depressive symptoms over time (Molnar et al., 2018). This model has been designated as a Best Practice by the Association of Maternal and Child Health Programs.

## Program Model

Project 1 and 2 LAUNCH and SOC were part of a statewide partnership between the Massachusetts Department of Public Health and the Boston Public Health Commission to improve ECMH in three cities in Massachusetts. The two programs provided a continuum of ECMH services in six primary care or community-based settings to young children (ages 0–8).The two programs served a diverse population in primarily low-income Latinx communities (N = 466 children), of which more than half were 5 years or younger at intake. LAUNCH provided services for families with young children (ages 0–8) to promote social emotional wellness and prevent poor mental health outcomes (Boston Public Health Commission, 2011). SOC provided services for families with young children (ages 0–6) who already have mental health needs meeting medical necessity criteria for intensive care coordination and wraparound services (Boston Public Health Commission, 2016). Both programs provided a continuum of mental health services from promotion and prevention to intervention.

The family partners in these models have lived experience navigating systems (e.g. health systems and education systems), accessing services to meet their own children’s ECMH needs, are trained in applying this experience to engage families and assist with systems navigation, and are often of similar racial/ethnic/linguistic and cultural backgrounds as they families they serve. The family partners in both programs were typically native Spanish speakers, were from the same communities as the patient populations that they served and had several years of experience working in family support services, early childhood, and/or the mental health field. Unlike other models wherein clinicians often supervise peer-support workers, in this model, the family partner and clinician work as equal partners with different but complementary skills (Boston Public Health Commission, 2014). The family partner and clinician teams work collaboratively with the families to develop care plans and set goals. The clinicians use their skill set in providing therapeutic services whereas the family partners use their expertise in engaging families, providing resources, and building skills.

The family partners and clinicians in both programs undergo extensive training in topics essential to ECMH through an established statewide learning collaborative. These learning collaboratives occur throughout the year and provide continuing education and training in the following topics: identifying and responding to ECMH risk factors; use of standardized screening and assessment tools to engage families; consistently considering the role of race, racism, and inequities; proactively coordinating with other service providers; maintaining healthy, empathic boundaries; using protocols to ensure physical and emotional safety of children, caregivers, and staff; and utilizing the expertise of lived experience to serve the caregiver-child dyad. Family partners also received ongoing reflective supervision at their sites to further refine their skills.

## Current Study

The purpose of this qualitative study is to explore the value of the family partner role in this model. This study is novel in two respects: (1) the focus on young children, many of whom were not yet enrolled in school and who were not yet formally enrolled in mental health services prior to participating in these programs, and (2) the inclusion of valuable first-hand perspectives of caregivers. Prior studies that have included caregiver perspectives focus on older children who are school-aged or on adolescents (Gyamfi et al., 2010; Jamison et al., 2017; Markoulakis et al., 2018; Reich et al., 2004). Including caregiver perspectives in evaluating service-delivery models for very young children is essential to develop a more nuanced understanding of the model’s strengths, and the most effective components to reduce barriers to engagement for families.

## Methods

### Participants

Nine leadership interviews were conducted with 12 participants from agencies that were a part of these programs: one from each of the three local health departments, one from each of the three community service agencies (CSAs) and one from each of the partnering primary care sites in SOC. Multiple leaders from the same agency could participate in the same interview. Individual interviews were also conducted with SOC staff: family partners (n = 2) and clinicians (n = 4), at each of the three SOC sites.

Staff from the LAUNCH sites (2 family partners, 3 clinicians, and 1 administrative coordinator) and SOC sites (3 family partners and 4 clinicians) participated in a separate focus group for each program. Two family partners and one clinician participated in both the SOC staff interviews and focus group.

The family partner/clinician teams identified participants for the caregiver semi-structured interviews at each site. Selection criteria for the caregivers was that they were families who had been actively receiving services for 3 months or longer. The 3-month cutoff was chosen to ensure that families sampled had been engaged with the team for a considerable amount of time and could therefore appropriately comment on their experiences. Two caregivers from each of the three LAUNCH sites and from two of the SOC sites participated (n = 10). The third SOC site was operating without a family partner for an extended period of time during the data collection period. As the focus of this work was on experiences with family partners, this site was excluded from recruiting caregivers for interviews since participants had not received any family partner services during that time.

**Fig. 1:**
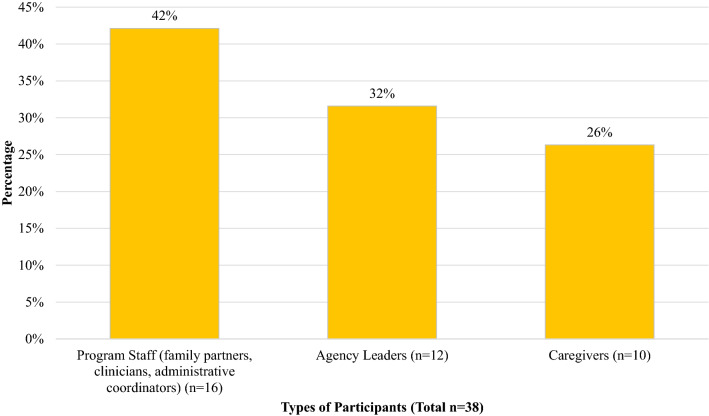
Types of participants

In total, there were 38 unique participants across the two focus groups and 22 interviews: 16 staff (family partners, clinicians, administrative coordinators), 12 agency leaders and 10 caregivers. A visual representation of the types of participants is presented in Fig. 1.

## Procedures

Research team members working in pairs under the direction of the Principal Investigator for the evaluation study conducted all interviews and focus groups. Interviews and focus groups were audio-recorded.

The individual interviews and focus groups with staff and leadership were semi-structured and included open-ended questions about the family partner. Specifically, participants were asked to describe the role of the family partner in the model and to describe the relationship between the family partner and the clinician in the model. Family partners and clinicians were also asked to comment on the impact of the family partner’s lived experience on service delivery. All staff and leadership interviews (n = 12) and focus groups (n = 2) were conducted in English.

The semi-structured individual interviews with caregivers also included open-ended questions about the role of the family partner in the care they received. Participants were asked to describe what the family partner did when they were enrolled in the program, whether and in what ways the family partner impacted their lives, whether working with the family partner changed their relationship with other providers including doctors and nurses at their primary care sites, and whether and in what ways the family partner’s services compared to other types of services they might have received. Interviews concluded with an open-ended question where participants were asked if they would like to share anything else about their experiences working with a family partner. Since the programs offered services in English and Spanish, and the families were primarily Latinx, caregiver interviews were offered in English and Spanish. Half of the caregiver interviews (n = 5) were conducted in Spanish with participants who were primarily Spanish speakers.

Following data collection, recordings were transcribed verbatim. Spanish interviews were conducted, translated into English and transcribed by a bilingual member of the research team. Three authors (SSN, CT, JW) independently analyzed the notes and transcriptions from all interview and focus groups using a six-phase thematic analysis approach in NVivo qualitative analysis software (Braun & Clarke, 2006). First, each researcher worked independently to generate preliminary codes. The research team then met to discuss these codes and then collated them into a set of themes through group discussion. The coding team met with the larger set of authors to discuss each theme and finalize them. This study was approved by the Northeastern University Institutional Review Board, the Massachusetts Department of Public Health Institutional Review Board, and the relevant sites. The authors declare that there are no known conflicts of interest and all authors certify responsibility for the contents of this manuscript.

## Results

Qualitative thematic analysis of the interviews and focus groups identified five overarching and interdependent themes that speak to the crucial role of the family partner in family-centered ECMH services: (1) the centrality of lived experience to the family partner role; (2) the importance of the family partner in family engagement and relationship building; (3) the value added by the family partner in navigating systems; (4) the ability of the family partner to build skills and empower caregivers; and (5) the role of the family partner in alleviating caregiver stress and other mental health concerns. Quotes are presented in-text with additional examples of exemplary quotes provided in Table 1.

**Table 1:** Additional exemplary example quotes

Theme	Quotes
*The centrality of lived experience to the family partner role*	“I feel that when the family partner shares pieces of her story, parents really connect and they don’t feel alone and they feel understood by their providers. I’ve heard a lot in the past from families coming to us and saying ‘they give me these strategies and expect it to work’ or expecting a mom of five being able to do something like this. That’s not something many providers think about but having a family partner you can consult with brings in that realness and perspective.” - Clinician
	“I do think lived experience is very important, so that will add to the credibility. It's not just a clinician telling the family, ‘This is the service that you need’ or ‘This is why you need to bring your children, or your child, to see the clinician.’ It's more about, ‘Okay, I know that you're maybe scared at this point and I know that this feels unknown, or I know that this is a challenge to you, based on our culture’… So I think that having that person there that has been through it and maybe can encourage them in a way that the clinician cannot, because they don't know all of those thoughts and feelings and maybe the uncertainty that surrounds the family.” - Agency Leader
*The importance of the family partner in family engagement and relationship-building*	“[The family partner] was, let’s say that she was there, she was always there. She made sure that I was comfortable, she made sure that I had everything all set, she made sure that I had [child] all set. She was mainly focused on [child]’s goals. She was very impacting, she will always be. I know that whoever gets her is very lucky.” - Caregiver
	“So, one of our family partners does amazing work out in the schools because she has been to Individualized Education Program meetings herself. She knows how scary it is to be around a table with 10 people, 15 people, 20 people, who knows how many people can be at those meetings. I think the way [family partners] bring their experiences to the table in truly a relationship-building way and not a ‘I know better’ way or a ‘let me tell you how I did it’ way, but truly a way to connect with families…and if your primary motivation, regardless of what your lived experience is, is to build that relationship, that’s what makes you successful in that role. And I think that’s what we have seen across our family partner role which has been wildly successful here based largely on staffing and who those individuals are.” - Agency Leader
*The value added by the family partner in navigating systems and building bridges*	“[The family partner] and I usually meet weekly. Anywhere from two to three hours, sometimes four hours at a time. We basically go over things that need to be done, like food pantries, appointments. Like coordinating all services you can think of, doctors’ appointments, things like health that I need, clothes, food. Even for myself going to school, college or whatever. Pretty much we cover all bases, it’s not just for [child], she does it for the whole family.” - Caregiver
	“Part of why we've had disparities is because there are whole groups of families out there who don't feel that the service provider system is the place to seek help from. And so, when you have a family partner who has sought help from that same system and can tell stories, both good and bad— about having had bad experiences, but they persevered, or they had good experiences and are good people out there, and I will help you find one— It bridges that gap and much like a cultural broker, it bridges that gap and I think allows a certain portion of those families that otherwise would stay away, in. And that's their biggest value.” - Agency Leader
*The ability of the family partner to build skills and empower caregivers*	“You are just like empowering the caregiver, and reminding the caregiver that they are decision makers, and also helping them with the other parties…Caregivers are always happy that you are pushing that even though sometimes when it comes to caregivers that want you to make the decision, that want you to take the lead. But when you help them gain that power back from some entity that made them feel disempowered, it really, it makes them feel grounded I think.” - Family Partner
	“[Family partner and clinician] actually gave me more support and …more faith in me, like okay, you’re gonna get this done, you know you’re gonna get this done…so it’s like, they didn’t doubt me. And eventually I did. I got my apartment, [my son] is in kindergarten, I’m trying to get [the baby] in daycare, hopefully I can go back to working soon.” -Caregiver
*The role of the family partner in alleviating caregiver stress and other mental health concerns*	“[Family partner] did help me get organized, like with my mindset, and try to focus straight because I have really bad anxiety….and my mind is just not all right there. So, she definitely helped me a lot with that.” - Caregiver
	“[W]hat our family partners do a wonderful job is not – Of course they’re going to make sure they’re helping people apply for a shelter and getting them all the applications that they need – but more importantly, they’re working with the families on the stress of being homeless. That’s not to say that our community health workers aren’t also doing that but our family partners, because of their training, because they work so closely with a clinician, because they are part of our behavioral health department functionally, they go about that work in such a different way, which I think makes their jobs better.” - Agency Leader

### The Centrality of Lived Experience to the Family Partner Role

Participants described the lived experience that family partners have navigating systems and accessing services to meet their own child’s ECMH needs as a central piece that lays the foundation for family partners’ interactions and engagement with families. This lived experience permeates every aspect of the family partner’s work with families, from engaging them in services to helping them with parenting challenges and systems navigation. Family partners described their role as different from other healthcare providers because their work is informed by their own life experience dealing with the same issues caregivers often face, and by understanding the challenges and emotions that caregivers might be going through. Family partners felt able to connect with caregivers as peers and discussed the importance of lived experience in allowing them to help families navigate housing, food, school systems, and healthcare systems. As one family partner explained,

My role, I feel like it’s different because in most of the things I help the family with, I’ve been through it or I’ve done it myself. So, when it comes to helping them navigate, for example the school system or food banks or anything like that, I can use my own experience to help them.

Unlike other providers, who might refer and connect families with services that they themselves may have never used, family partners are able to do this while highlighting their own past experiences working with these services.

In addition to helping caregivers navigate complex systems, family partners often share their own stories and help caregivers feel less alone in the process of raising a child with mental health challenges. Family partners and clinicians described lived experience as being the foundational element that allows the family partner to reduce caregivers’ sense of isolation, frustration, and anger. Lived experience helps family partners create an authentic relationship based on mutual understanding and empathy. As one family partner put it,

I understand where they are coming from, I have been there. I have been frustrated with the school system, I have been frustrated with the medical services because I sometimes say wait- I understand where this is coming from, I could understand you in seconds, you are being upset, you are irritated, and I can feel it…I try to meet them where they are and then being there for them.

### The Importance of the Family Partner in Family Engagement and Relationship-Building

Family partners, clinicians, agency leadership and caregivers collectively described the critical role the family partner plays in engaging families in services at the onset, and then forging lasting and meaningful relationships that keep families engaged in services. Family partners described leveraging their unique, lived experience by using intimate knowledge of resources and parenting practices in the family's communities to discuss various strategies for working with children and caregivers. This connectedness to the community allows family partners to engage caregivers in a way that the clinicians are often unable to offer. As one clinician shared,

[A] mother was very depressed and couldn’t get herself to medical appointments. She was re-referred to us and completed intake after meeting with her behavioral health provider. She shared a very challenging story that she’s going through and [family partner] shared her own story. When [the mother] came back for her next visit she was happy and well-dressed. Her energy level had changed, and she told me that after the last visit she left very motivated and bought a white erase board and did a calendar. We didn’t tell her to do that, but it was something she wanted to do to help organize herself, and she did little chore charts for the kids. I don’t know if it was that or a combination of other things, but it was great to hear that she felt good after she left our visit and I’m sure it was because [family partner] was able to share her piece of it.

In this narrative, the family partner was able to engage the caregiver in services, and help the caregiver feel motivated and energized, by sharing her own lived experience and connecting with the caregiver on a personal level.

Caregivers described the family partner as an individual they could depend on and develop a different relationship with than with traditional providers. By being available in situations of crises and high-stress, family partners are able to make parents feel they are well-supported by their providers. As one caregiver put it,

My son landed in the hospital right before Christmas…that was a tough time especially being around the holiday. The way it happened [family partner] was really a support calling every day, she was coming in to see us making sure I wasn’t alone, she was making sure I was eating… pretty much just being there as a support. It’s almost like [family partner and clinician] become more than a team; it’s almost like a family. They’re there whenever you need them.

One of the key ways in which family partners create relationships with caregivers is by proactively checking on them through phone calls, text messages, and frequent in-person meetings. By keeping in frequent contact with caregivers, family partners are able to develop trusting relationships and help caregivers feel valued by their providers. This sentiment that family partners are always there for caregivers is a recurring theme that echoed across interviews. Many caregivers felt that this regular availability was an important part of the family partner/caregiver relationship, and one that caregivers valued immensely. Caregivers expressed feeling deeply impacted by the work of the family partner.

As one caregiver shared,

I am very thankful to the two of them, but [family partner] was always checking. All the time. She would call me, and ask, do you have food? And then she would take me to the pantry, and we would find food.

Relationship-building by family partners often extended beyond the clinic or the home. Family partners frequently met with caregivers in different locations, a strategy which counters social isolation. As one caregiver said, “She was really, really good with [child] as well; we did a lot of meetings at other places besides my house. So, we did go out, we didn’t stay at my house all the time.”

### The Value Added by the Family Partner in Navigating Systems and Building Bridges

Family partners, clinicians, agency leadership and caregivers all described one of the key roles of the family partner as helping families navigate multiple systems and agencies to obtain services and resources. While physical health, mental health, housing and school services were mentioned by most caregivers, some caregivers also described the importance of accessing food, clothing, and legal services. Some caregivers also spoke about the importance of being able to attend social events in the community with their children and the receipt of practical resources such as backpacks, school supplies and holiday gifts for their children. As family partners helped families navigate systems, they served as a bridge between caregivers and these various services and agencies that might were otherwise unfamiliar to them. This can also help address disparities for families by engaging them within these systems and improving the quality of services.

Family partners’ lived experience and assistance in navigating school systems and policies, housing authority processes, and child welfare agency rules and regulations were an important asset to caregivers. Family partners shared their own daunting experiences negotiating with school department professionals to find a placement for their child or develop an Individualized Education Program (IEP), which is even more challenging when a child has already been sent home multiple times for behavioral issues. One caregiver described the family partner as an advocate,

I was struggling with the school system…and I wasn’t getting anywhere…I kept saying it wasn’t the placement he needed and the school kept playing around with it….[then the family partner] called the school, they were there at meetings, they were pushing, [they] called the head school personnel…and got them to go in and do an evaluation for him to get him put in a different placement.

Family partners also help caregivers navigate the health and mental health care systems. They can explore practical obstacles to following through on referrals (e.g. lack of transportation or childcare), or explore and address a family’s ambivalence or reluctance to follow through on a referral. Often, family partners accompany families to referral services if needed. A caregiver reported, “[Family partner] once went to an appointment with me and my son. Cause I was stuck on it, like where to go and stuff. And she actually went and was there as a support.”

In addition to helping caregivers follow through on referrals, family partners who speak the same language as families can bridge linguistic challenges families face in systems navigation. This was especially important in our sample of Latinx families, many of whom were primarily Spanish speakers. Likewise, family partners can help bridge the gap between caregivers and health care professionals by explaining complex health and behavioral health care symptoms, diagnoses, and instructions in language that is understandable to non-clinicians. One caregiver shared,

But if I need help, I’ll be like [family partner] can you come help me do this real quick? Or explain it to me in a way that I can understand, and she’ll explain it to me. Cause sometimes I don’t understand things cause you know how everyone’s doing professional talk?… So most of the time, I don’t understand it. So, I’ll call her up and say, ‘they said this, can you explain it to me so I understand, cause if not, I’ll take it wrong and then I’ll start panicking.’ And she’ll say, ‘no, this is how they said it, this is what’s going on. You’ll be fine.’ And I’m like (sighs) okay.

Another caregiver recounted,

[Family partner and clinician] go to the doctors, referrals, and everything that has to do with my daughter, like school, neurologist, doctors…since I don’t understand English well, and sometimes the documents come in Spanish or English, and I don’t understand English clearly. Well, I go with [family partner], or give [family partner] a call. Or when my daughter and I have an appointment. She helps me. She provides me with that service.

In helping families access services, family partners often serve as an advocate, role model or cultural broker in interactions with service professionals. As one agency leader shared,

[B]ut really what it comes down to, is having to navigate various systems within the system of care, whether it be school, a Department of Children and Families, all the way down, and is not only understanding how that works, but also having a lived experience that can connect with the parent on what it's like to be a parent with a child with these needs. Which kind of really is an added benefit to the clinician who is going to be speaking to this parent, as there's a hierarchy issue that people sometimes worry about, or that the clinician may have gone to school, learned a lot about that experience working in the field with what the parents are dealing with, but not knowing what it's like to actually be a parent in that role. So, this kind of helps with that.

### The Ability of the Family Partner to Build Skills and Empower Caregivers

A prominent finding from the caregiver interviews is the important role family partners played in helping caregivers build skills for parenting their children, interacting with other providers, and managing their daily lives. Caregivers reported sometimes being challenged to find appropriate responses for their child’s behaviors. Family partners draw on their own experiences to model what a path forward might look like if the caregiver is struggling with aggressive behaviors, temper tantrums, bedtime routines, food choices and homework. Family partners provide mentoring and coaching in these areas to foster skill-building. One family partner summarized it by saying, “As a family partner what I do is educate, counsel and model. And within weeks, a certain goal already accomplished because of those three steps.”

Caregivers described multiple examples of family partners providing suggestions regarding child behavior, with a particular emphasis on positive reinforcement. Furthermore, family partners also empowered caregivers with knowledge that they could apply in different domains of their lives. One caregiver recounted,

I’ve been a parent for twenty seven years, but there’s a lot of things that I didn’t know….so instead of yelling and saying “hey, put that down!’ there’s different ways of doing it….try to encourage [her daughter], try to tell her ‘oh, you’re doing a great job, good girl,’ when she does something, You know, praise her so she feels confident….[family partner] taught me how to really talk to her calmly and how to deal with her if I get so mad or angry or something.

Another area of skill-building important to caregivers is around decision-making. Caregivers, clinicians, agency leaders, and family partners talked about the importance of ensuring that caregivers are the decision-makers and are setting their own goals for the child. Family partners can act as allies helping caregivers assume a decision-making role in their lives and in addressing their children’s needs. Rather than giving advice or instructions, as many professionals do, family partners provide options, choices, and develop shared caregiver-driven goals. As one agency leader said,

[Family partners] don't try to give advice. Like, pediatricians love to give advice and tell parents what to do. I think that they probably have the skillset where they helped develop a shared, help the parent identify goals for the child that the parent sees as their goals.

This approach builds caregiver confidence and hope, and can be very empowering. One family partner told us that they tell caregivers, “It’s, we’re not the experts of your child, you are…our process really helps with what we stand for, family voice and choice.”

Many caregivers described learning about and using organizational tools to structure their own lives as well as those of their children. While the family partners, clinicians, agency leaders, and the caregivers talked at length about developing new parenting behaviors and empowerment, only the caregivers spoke about the importance of how family partners provided them with concrete tools to structure and guide the activities of daily life. One caregiver shared,

[Family Partner] taught me a bunch of stuff. Like, stuff I didn’t even know. She taught me, how to organize…how to do a portfolio… She made a nice binder for me and it had the scheduling of the school year, it had the days in the month and stuff like that.

### The Role of the Family Partner in Alleviating Caregiver Stress and Other Mental Health Concerns

A major impact of the family partner role is the alleviation of stress in caregivers’ lives. The family partner not only meets immediate needs and facilitates resource connection, they help families cope with the stress of having unmet needs and a lack of resources. For example, as one agency leader described, while any provider may provide information for families on potty training a toddler, a family partner may provide reassurance in supporting a family living in a shelter with a communal bathroom, by recalling their own stress (and success) with potty training in a similar situation. Caregivers spoke explicitly about ways that the family partners helped them to manage and address their stress, and other mental health concerns, including anxiety, depression and anger management. One caregiver shared,

[The family partner] helped me so much…because I felt very depressed. So much depression that when I remember, I feel like my body is shaking…she helped me to move through it and she used to tell me ‘you’ve got to be strong’ because I would start shaking.

Caregivers also spoke about how the family partners helped them resolve challenges that other family members such as the caregivers’ parents, partners or other children were facing, that added stress to the caregivers’ lives. Program staff also indicated that this model allowed them to address the entire family unit by assessing and caring for both the caregiver and the child in a comprehensive manner. One caregiver reported,

My mother needed support…I needed to know where could I go to help my mother. Because, since the team and I arrived to the conclusion that, the less stress, even in situations that are outside the house… so as [family partner] helped me to find the resources… All of that helped me to reduce my stress, and to have extra time for my kids.

Family partners also worked with caregivers to take care of themselves and provide strategies for self-care. Caregivers described the family partner as working with them, supporting them to better care for their children. As one caregiver said,

Like I was having a situation with my kid’s father, and stuff like that. So, she was telling me, if you’re not happy, you don’t have to be there. If you’re happy, make it work. You know, do for the kids, make sure the kids are happy and stuff. And I said, I am. I’m trying to make sure the kids are good, my priority first is always to try to make sure they’re good before myself. And she said, it’s good to do that, but I also got to take of myself in order to take care of them. And I said, you’re right… So I’m trying to balance it a little bit.

Another caregiver described,

Well, his in-home therapist would work with him, and [family partner] would work with me, well, she also worked with him. It was kind of a family thing, but she mostly worked with me so I could do what I have to do, you know, to get him situated as well.

## Discussion

The present study adds to a growing body of literature on the value and importance of peer support workers in delivering care to families of children with behavioral health needs. Our findings indicate that family partners are in a unique position to leverage their lived experience and professional training to provide high-quality family-centered care engaging caregivers and their children in services, and directly addressing various social determinants of health. Caregivers receiving care through the family partner-clinician model in this sample reported reduced stress and other mental health concerns, enhanced capacity for communication, increased feelings of empowerment, and improved self-care. This is consistent with findings from studies with older children (Brister et al., 2012; Ireys et al., 2001; Radigan et al., 2014).

There are multiple barriers to engagement for families accessing ECMH care at both the individual and community levels. As a result, disengagement is high—estimated to be between 20% and 80% across studies (Ingoldsby, 2010; McKay & Bannon Jr., 2004). Participants in this study highlighted the importance of the family partners in both engaging new families into care, and also in motivating families to remain engaged in care for a longer period of time by addressing barriers at multiple levels. Family partners were able to counteract practical and logistic barriers such as language accessibility, health literacy, transportation issues, school issues, housing concerns and food insecurity. In these programs, the family partners shared cultural, racial, and/or linguistic backgrounds with a majority of the families who were Latinx Spanish speakers. This was especially important in navigating language barriers for families who primarily spoke Spanish. In addition, they were able to address psychological and cultural barriers including fear, stigma, labeling, distrust of clinicians, and self-blame on the part of families (Brauner & Stephens, 2006; Ingoldsby, 2010; Nelson & Mann, 2011). Dismantling these structural barriers for families of young children seeking ECMH services, especially those from communities suffering from economic hardships and social stress, is essential to improving health equity and improving child and caregiver outcomes. Our findings demonstrate that the inclusion of a family partner, with lived experience navigating systems and accessing services to meet their own child’s ECMH needs, is a feasible and highly regarded solution for breaking down some of these systemic barriers.

Moreover, family partners are able to engage families in ways that the traditional medical hierarchy usually cannot. Family partners cultivate meaningful relationships with families, by sharing their personal stories and experiences, modeling productive problem-solving strategies, connecting families to valuable resources, encouraging families to become decision-makers in the care of their children and themselves, and being consistently available and supportive. The lived experience that family partners bring with them to this work is key in empowering caregivers through partnerships rather than power-imbalanced patient-provider relationships. They provide services in a way that is seen as non-judgmental by families because they themselves have been in similar situations to those they are helping families with. They are able to share their personal stories with intent, and to impart hope to caregivers who are facing extremely challenging circumstances. Sharing lived experience in service delivery has potential to truly engage families in a continuum of care, and in the LAUNCH/MYCHILD model of ECMH integration family partners are trained and supported in using their experience with intention while maintaining professional boundaries. It is the family partners’ lived experience that is seen by program staff and leadership as essential to this engagement, and that sets family partners apart from traditional community health workers.

Our findings add to the literature on interventions that promote positive parenting practices and parent training to manage child behavior in children with behavior problems, including models such as the Parent Management Training—Oregon Model (Forgatch & Kjøbli, 2016; McIntyre, 2013). In particular, mentoring caregivers and building parenting skills are key components of family support, and are greatly appreciated by caregivers. Family partners in our study provided and coached caregivers to use organizational tools for managing day-to-day activities to alleviate parenting challenges. Helping caregivers organize and structure their daily lives is often discussed as part of strengthening caregiver competence in caregiver literature focusing on family members with chronic illnesses or disabilities (Reinhard et al., 2008), and we find that these are important aspects of service delivery to support those caring for young children. When organizational tools are shared in the context of the family partner’s lived experience, they strongly resonate with caregivers. By modeling parenting practices and assisting caregivers with organization strategies, family partners embody a family-centered approach that works to ensure long-term success in creating a positive home environment for young children, including those with social, emotional and behavioral health needs. Family-centered approaches are essential as they have been associated with better mental health outcomes for children (Burak & Rolfes-Haase, 2018; Holcomb-McCoy & Bryan, 2010). Moreover, family-centered care empowers families through shared decision-making that creates an authentic partnership between providers and clients (Kuo et al., 2012).

Consistent with this family-centered approach, when planning interventions to address ECMH needs it is important to identify and address caregiver mental health needs that impact their ability to parent and access services for their children (Fawley-King et al., 2013; Staudt, 2007). Across interviews, participants in the study reported the invaluable role that the family partner played in helping caregivers manage their own mental health challenges. Caregivers indicated that family partners helped them improve their own mental health and provided them with concrete skills and tactics to use in times of crises and high-stress. Empowering families to improve their self-efficacy and confidence is a core component of the work family partners do, which is deeply informed by their experience navigating and overcoming similar challenges in their own lives. As previously mentioned, the quantitative evaluation of the original LAUNCH project found that depressive symptoms and caregiver-related stress decreased across the year of follow-up (Molnar et al., 2018).

The role of the family partner is particularly suited to working with families facing a variety of structural and systemic barriers to care, who might not otherwise be engaged in services for their children with social, emotional and behavioral health needs. By engaging these families with young children and assisting with navigating systems and barriers, the family partner-clinician team is able to intervene and mitigate problems earlier, preventing long-term negative impacts to mental health and wellness. By helping to directly address multiple social determinants of health, the family partner approach may be a model that could serve to reduce health inequities.

### Limitations

Findings should be interpreted in the context of study limitations. The overall sample size of caregivers for this study was small (n = 10) and thus these experiences might not be generalizable to caregivers of young children in other contexts or locations. Given the small sample size of families who did not share cultural and/or linguistic backgrounds with the family partners in the programs, we could not assess in what ways the experiences of families would be different if their family partners were of a different cultural/racial/ethnic background. Moreover, we used a non-probability purposive sampling scheme for the caregiver interviews. As a result, we cannot compare our findings to families who have received similar services without a family partner, nor can we compare to families who disengaged immediately to examine factors that limited their engagement in this model. However, themes identified by caregivers were also corroborated by other types of participants which increase the robustness of the findings.

## Conclusions and Future Directions

The findings from this study indicate that family partners with lived experience play a key role in engaging families in mental health services by using their lived experience to build rapport and help families navigate services and build skills. As a result, families engaged with the family partner-clinician dyad reported an alleviation of stress and other mental health concerns. The early engagement and effective services that family partners deliver are a promising strategy deserving of more policy attention, including developing payment mechanisms for teaming of integrated family partners and ECMH clinicians in primary and community-based settings. Delivering comprehensive, two-generational services that provide care for caregivers and children in a coordinated manner will result in improving the quality of services delivered and received. Additional large-scale research examining the effectiveness of family partners in the provision of mental health services for very young children and their families is warranted.
